# The Importance of Post-Inflammatory Polyps (PIPs) in Colorectal Cancer Surveillance in Inflammatory Bowel Diseases

**DOI:** 10.3390/jcm14020333

**Published:** 2025-01-08

**Authors:** Ivanna Candel, Panu Wetwittayakhlang, Talat Bessissow, Peter L. Lakatos

**Affiliations:** 1 Division of Gastroenterology and Hepatology, McGill University Health Centre, Montreal, QC H3G 1A4, Canada; wet.panu@gmail.com (P.W.); talat.bessissow@mcgill.ca (T.B.); 2 Gastroenterology and Hepatology Unit, Division of Internal Medicine, Faculty of Medicine, Prince of Songkla University, Songkhla 90110, Thailand; 3 Department of Internal Medicine and Oncology, Semmelweis University, 1085 Budapest, Hungary

**Keywords:** pseudopolyps, post-inflammatory polyps, inflammatory polyps, inflammatory bowel disease, ulcerative colitis, colorectal neoplasia, colorectal cancer

## Abstract

Inflammatory bowel diseases (IBDs), encompassing Ulcerative Colitis (UC) and Crohn’s Disease (CD), are chronic inflammatory disorders affecting the gastrointestinal tract. The association between IBD and colorectal cancer (CRC) is well-documented. Multiple factors have been identified as contributors to the risk of developing CRC in patients with IBD, including duration of disease, disease extension, family history of CRC, co-existance of primary sclerosing cholangitis (PSC), and potentially the presence of post-inflammatory polyps (PIPs). PIPs, often referred to as pseudopolyps, are polypoid structures that emerge as a result of severe mucosal inflammation. While their presence has been linked to greater disease severity, the role of PIPs in increasing CRC risk remains controversial. Increasing evidence suggests an association between post-inflammatory polyps (PIPs) and the risk of colorectal neoplasia, with PIPs potentially serving as an indicator of this risk through a history of enhanced inflammation. PIPs may also be linked to a distinct patient phenotype, including the presence of other known risk factors. More recent studies suggest that the risk burden (characterized by a high number or by large polyps) may be important. However, the evidence remains inconsistent, with some studies showing no clear association between PIPs and CRC risk after adjusting for other factors, including histological inflammation. In contrast, the data suggest a low rate of malignant transformation of the PIPs themselves. This narrative review aims to summarize the latest evidence regarding the relationship between PIPs and CRC in IBD, with a focus on UC. While some studies suggest that PIPs may serve as markers of higher disease severity and inflammation, their direct contribution to CRC risk remains unclear. Further research is needed to explore the inflammatory and carcinogenic pathways in patients with PIPs to better understand their role in colorectal cancer (CRC) development.

## 1. Introduction

Inflammatory bowel diseases (IBD), encompassing Ulcerative Colitis (UC) and Crohn’s Disease (CD), are chronic inflammatory disorders affecting the gastrointestinal (GI) tract, characterized by periods of relapses and remission. The highest prevalence of IBD worldwide has been reported in Western Europe and Canada, while Asia exhibits a lower but steadily rising prevalence. Studies examining temporal trends indicate that the incidence of IBD continues to grow in many regions globally. As a result, IBD is emerging as a global health issue, placing an increasing burden on healthcare systems [1,2].

The link between IBD and CRC is well established; however, reported risk estimates show considerable variation [3]. Patients with colonic inflammatory bowel disease have a 1.7-fold higher risk of developing CRC [4], with some recent data showing a decrease from 1.34 in 1979–1988 to 0.57 in 1999–2008 [5]. In patients with UC, the risk of CRC is elevated, with reported incidence rates varying from 3 to 20 times higher than those observed in the general population [6,7,8]. According to Askling et al., the absolute risk of CRC after 30 years of follow-up in patients with pancolitis was 11% for those diagnosed with pancolitis before the age of 25. This is similar to the Uppsala cohort, where the risk was 13% in patients with pancolitis diagnosed before the age of 40 [6,9].

According to Lutgens et al., in a meta-analysis, the pooled standardized incidence ratio of CRC in all patients with IBD in population-based studies was 1.7 (95% confidence interval, 1.2–2.2). The cumulative risks of colorectal cancer (CRC) were 1%, 2%, and 5% after 10, 20, and more than 20 years of disease duration, respectively. Between 1990 and 2010, the risk of colorectal cancer in people with UC showed a declining trend in some geographic regions. The declining risk of colorectal cancer in people with UC may be explained by advances in treatment of UC and the implementation of dysplasia surveillance programs [4,10]. Cumulative risks of CRC in the general IBD population are also elevated, although to a lesser extent than those previously reported by Eaden et al. [11].

Regarding clinical and histological risk factors, such as extensive colitis, family history of CRC, concurrent primary sclerosing cholangitis, or PIPs [12,13,14,15], patients with IBD are categorized into different risk categories for surveillance with some differences between American and European guidelines [16,17]. The American guidelines recommend surveillance colonoscopies every 1 to 3 years [16,18,19], while the European guidelines (ECCO, BSG) stratify patients into risk categories to determine appropriate surveillance intervals [12,17].

Numerous risk factors for IBD-associated colorectal cancer (CRC) have been identified, necessitating intensified surveillance strategies for affected patients. In addition to disease duration, several factors increase the risk of colorectal cancer (CRC) in IBD patients, including the extent of colitis, family history of CRC, previous dysplasia, co-existing PSC, and post-inflammatory polyps (PIPs). Ekbom’s analysis reported standardized incidence ratios (SIR) of 1.7 for proctitis, 2.8 for left-sided colitis, and 14.8 for pancolitis compared to the general population [9]. More recent studies suggest a lower risk, with SIRs of 5.6 for pancolitis, 2.1 for Crohn’s colitis, and 1.7 for proctitis [20]. Case–control and population-based studies show a 2–3-fold increased risk in patients with a family history of CRC [21]. Askling et al. found a 2.5-fold relative risk (RR) for IBD-related CRC (95% CI: 1.4–4.4), and that those with a first-degree relative diagnosed before age 50 had a significantly higher risk (RR: 9.2; 95% CI: 3.7–23) [6]. Velayos et al. also identified family history as an independent risk factor for IBD-related CRC in patients with UC (OR: 3.7; 95% CI: 1.0–13.2) [22]. Soetikno’s meta-analysis of 11 studies found that UC patients with PSC have a significantly higher CRC risk (OR: 4.09; 95% CI: 2.89–5.76) [23]. PIPs are also linked to elevated CRC risk. These findings highlight the need for tailored and intensified surveillance in high-risk IBD patients.

**Figure 1:**
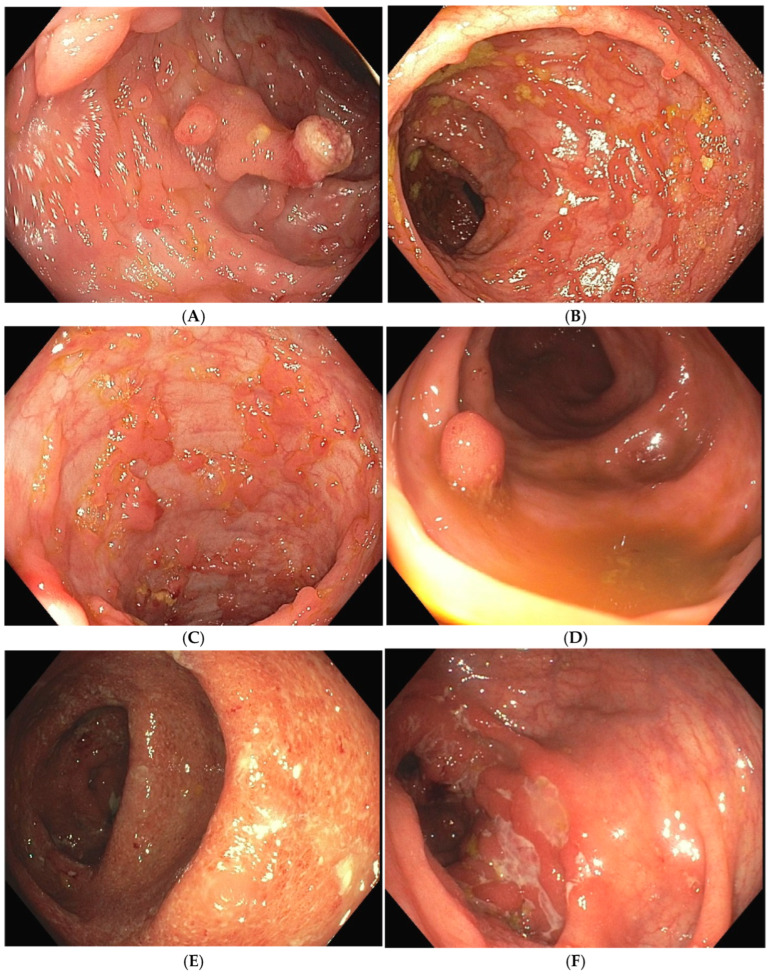
Endoscopic appearance of post inflammatory polyps (PIPs) (**A**–**C**) and true adenomatous polyp (**D**) in patients with ulcerative colitis, endoscopically active ulcerative colitis (**E**), and Crohn’s disease (**F**).

Post-inflammatory polyps (PIPs), often referred to as “pseudopolyps”, are polyp-like structures that protrude above the mucosal surface but do not possess malignant potential [24]. Figure 1 highlights the endoscopic presentation of PIPs, other polyps in IBD patients, and active inflammation of patients with UC or CD. PIPs occur in 10–20% of the patients with IBD [25]. They complicate UC twice as frequently as colonic CD [26,27], but can also be present in other scenarios [28,29].

Case–control studies have indicated that the presence of PIPs, which serve as markers of previous severe inflammation, is associated with an increased risk of CRC in UC [22,30,31]. Their number may vary widely per patient from few to innumerable. Their presence has, therefore, been suggested to be associated with greater risk for CRC as well. However, data from the published literature are conflicting. For instance, a retrospective multicenter cohort study involving 462 IBD patients with PIPs confirmed their association with greater severity, the extent of colonic inflammation, and an increased risk of colectomy, but did not find an elevated risk of CRC [32].

Although the presence of PIPs has been suggested to be associated with a high risk of CRC, it is questionable whether PIPs themselves denote a CRC risk and if they should be a subject of surveillance. This narrative review aims to summarize the most recent evidence regarding the relationship between PIPs and the risk of CRC. This review seeks to determine whether PIPs contribute significantly to CRC risk and to evaluate their role as a critical factor in surveillance strategies. Emphasizing this connection is essential for guiding risk stratification and optimizing surveillance protocols in patients with inflammatory bowel disease. Nevertheless, there is some controversy regarding the necessity of more frequent surveillance for IBD patients with PIPs [33].

## 2. Epidemiology

The reported prevalence of PIPs is estimated to be 10–40% in patients, with a broad variation in the published cohorts [26,30,34,35,36,37,38,39,40,41,42,43,44,45,46]. There is limited direct evidence linking PIPs and “burned-out colon”. In a retrospective cohort of 399 UC patients, the peak incidence for PIPs occurred in patients in their 30–40s [36]. The duration of the disease may be also relevant, although PIPs have been reported within months of the onset of colonic inflammation [47]. Teage et al. showed that PIPs were more prevalent in cases of severe total colitis, though they can also be found in left-sided disease, typically following a particularly severe relapse [42]. PIPs are found more often in UC than in CD, with rates of up to 2-fold more in some studies; they were significantly more common in patients with UC involving the entire colon [36] with a predilection for the transverse and left colon [36,48,49,50,51], and the incidence was similar in both sexes [36,52].

In addition, PIPs can lead to complications such as colonic obstruction, intussusception, bleeding, iron-deficiency anemia, and protein-losing enteropathy, along with concerns regarding the potential development of neoplasia, and therefore their endoscopic removal or surgery (colectomy) may be necessary in selected cases [53,54,55,56,57].

## 3. Association of PIPs with CRC Risk in UC

PIPs are visible markers of severe inflammation on endoscopy, and it has been suggested that the presence of PIPs is a risk factor for inflammation and CRC development [22,35,58,59,60].

Of note, a recent European retrospective multicenter cohort study found that patients with a high burden of PIPs, defined by the number and/or size of polyps, required more frequent treatment escalation, hospitalizations, or IBD-related surgery compared to patients with a low PIP burden. Although this study CRC was not investigated, one can hypothesize that the higher inflammatory burden (identified as the degree of inflammation and mucosal damage) can lead to an increased risk of carcinogenesis. Therefore, patients with a high burden of PIPs may be at an increased risk of CRC development compared to those with no or a low PIP burden. This suggests that a detailed assessment of PIP burden could serve as a more accurate indicator of inflammation severity in IBD than merely identifying the presence or absence of PIPs. Consequently, categorizing all patients with PIPs into the intermediate-risk surveillance group without evaluating the true PIP burden may be inappropriate [61].

Similarly, in a recent study by Wolf A. et al., in 2023, the relationship between PIPs, histological activity, and the risk of colorectal neoplasia (CRN) in IBD patients was examined. The study included 173 IBD patients, with PIPs identified during index colonoscopy and at least two surveillance colonoscopies, compared to 252 patients without PIPs. The time to advanced CRN development did not differ significantly between the two groups, and PIPs did not independently increase the risk of advanced CRN (adjusted HR: 1.17; 95% CI: 0.59–2.31). The presence of PIPs at index colonoscopy had no impact on CRN risk in patients with (*p* = 0.83) or without (*p* = 0.98) histological inflammation. Instead, CRN risk was strongly associated with factors such as active histological inflammation, a high Nancy index score of 3 or 4 (HR 3.44; 95% CI: 1.63–7.24), age (HR per 10-year increase: 1.37; 95% CI: 1.13–1.66), and a first-degree family history of CRC (HR 5.87; 95% CI: 1.31–26.26). After adjusting for histologic activity, PIPs did not increase the risk of CRN in IBD patients. This suggests that histologic activity, rather than the presence of PIPs, should be prioritized in CRN risk assessments [62].

Thus, the study concluded that PIPs are not independent risk factors for CRN but rather markers of histological disease activity (inflammation), which remains a well-established independent risk factor for colorectal neoplasia in IBD patients [62].

Current evidence suggests that both PIPs and CRN may be consequences of chronic mucosal inflammation, and PIPs are often associated with more severe inflammation, extensive disease, and higher colectomy rates [12]. The risk of CRC is elevated in IBD patients with chronic mucosal inflammation and extensive colitis [63,64].

Many studies identified the presence of PIPs as a risk factor for the development of CRN and CRC in IBD. In a case–control study, it was found that patients with CRC were significantly more likely to have PIPs (OR 2.4) during routine colonoscopy surveillance, remaining highly significant on multivariate analysis [30]. On the flip side, PIPs have been reported as a significant risk factor for developing CRN and CRC in patients with IBD. Earlier case–control studies identified PIPs as an important factor associated with an increased risk of CRC, with one study reporting an odds ratio (OR) of 2.5 (95% CI: 1.4–4.6) for CRC in patients with PIPs. Another study further supported this association, demonstrating a relative risk (RR) of 1.92 (95% CI: 1.28–2.88) for CRC in IBD patients with PIPs [31]. The association was not confirmed in all studies; e.g., a 2019 retrospective multicenter study by Mahmoud et al. found no significant difference in the development of advanced CRN between patients with and without PIPs (adjusted HR 1.17; 95% CI, 0.59–2.31) over a median follow-up of 4.8 years [32]. Similarly, after adjusting for confounders, the presence of PIPs was not linked to an increased risk of CRN (HR 1.28; 95% CI, 0.85–1.93) or advanced neoplasia (HR 1.38; 95% CI, 0.52–3.68) in another study [65]. In fact, approximately 50% of the studies reported a lack of direct association.

A recent meta-analysis of case–control and cohort studies revealed that 28.4% of IBD patients with PIPs were diagnosed with colorectal neoplasia, compared to 15.7% of IBD patients without PIPs. IBD patients with PIPs were significantly more likely to develop colorectal neoplasia (CRN, advanced CRN, or CRC) than those without PIPs (RR = 1.74, 95% CI: 1.35–2.24). Furthermore, PIPs were also significantly associated with a higher risk of colorectal neoplasia in UC patients (RR = 1.76, 95% CI: 1.18–2.63, *p* = 0.006) [33]. However, the included studies were enriched in patients with extensive colitis (30–100%) with a relatively high prevalence of PSC 4.1–13.3% and an average disease duration of 13 years. The overall quality of evidence, however, was rated as moderate to low. Moreover, four out of the nine included studies did not identify PIPs as a risk factor for CRN/CRC. In the study by Shi et al., 26.8% of IBD patients with PIPs were diagnosed with advanced CRN, compared to 10.2% of those without PIPs (RR = 2.07, 95% CI: 1.49–2.87). Additionally, the presence of PIPs was associated with an increased risk of CRC in IBD patients (RR = 1.93, 95% CI: 1.32–2.82) [33]. The summary of the studies investigating the association between the presence of PIPs and the risk of CRC is presented in Table 1 modified after Shi et al. [33].

In line with the above, when risk factors were assessed in IBD patients with CRC, in a recent systematic review by Wijnards A.M. et al. in 2021, PIPs were identified as risk factor for CRC in univariate analysis (OR 3.29; 95% CI, 2.41–4.48); however, this association was not statistically significant in pooled HR analyses, with univariable HR at 1.67 (95% CI, 0.99–2.82) and multivariable HR at 1.73 (95% CI, 0.88–3.40) [63]. Other factors, such as extensive colitis, low-grade dysplasia, intestinal strictures, PSC, and a family history of CRC, may play a more significant role in the development of CRN in IBD patients [63].

## 4. Do PIPs Turn into Cancer? Do We Need to Surveil the PIPs?

It is “historically” well established that PIPs do not undergo malignant transformation. Instead, they are considered surrogate markers for previous severe inflammation, which is believed to be the underlying risk factor for neoplasia [66,67]. For some of the PIPs, playing a role as surrogates of inflammation, there is also concern about the burden and the impossibility of finding dysplastic lesions in endoscopic surveillance [24].

The data assessing the risk of carcinogenesis in the PIPs, apart from case reports, are scarce [56,68,69]. However, in a very recent retrospective study authors identified dysplastic foci in 22% of the PIPs analyzed in patients with long standing IBD [70]. A multivariate analysis identified patient age (OR; 1.1, 95% CI: 1.02–1.12), lesion size (OR; 1.39, 95% CI: 1.15–1.68), and right colonic location (OR; 5.32, 95% CI: 1.01–26.9) as independent predictors of dysplasia. In a study by De Cristofaro et al., PIPs larger than 5 mm demonstrated a sensitivity of 87% and specificity of 63% for detecting dysplasia. Notably, nearly half of the dysplastic lesions in this study were found in hyperplastic polyps. However, the degree of dysplasia (high or low grade) was not classified in the study [70].

This raises the question of whether colorectal neoplasia surveillance is necessary for patients with PIPs. While there is growing concern that PIPs may be linked to an increased risk of CRN, current evidence remains weak regarding their direct contributions to the CRC burden in IBD patients.

Moreover, dysplasia surveillance of PIPs is challenging, resource-intensive, and time-consuming. Instead, intensive CRC screening should be prioritized for IBD patients also presenting with other risk factors such as extensive colitis, long-standing disease, coexisting PSC, or a family history of CRC, rather than solely the presence of PIPs.

## 5. Expert Opinion on the Importance of PIPs in UC

It is increasingly clear that PIPs are associated with the overall risk of CRN in IBD. However, they are more like an important indicator of chronic inflammation and are associated with a distinct patient phenotype, as seen in Table 2. In recent years, accumulating data have confirmed that PIPs alone may not pose a clinically relevant risk for advanced CRN in IBD patients. Consequently, the presence of PIPs alone may not require an intensified surveillance strategy for CRC screening. Of note, some studies reporting a positive association between PIPs and CRN or CRC did not adequately control for confounding factors—such as the degree of colonic and histological inflammation and other individual CRC risk factors—that could significantly impact CRC development, or these factors were more frequent in the PIP cohort compared to the general IBD patients.

In contrast, recent data suggest that the PIP “burden” (numbers: few vs. numerous and size: large), rather, should be considered when evaluating their potential contribution to CRC; thus, one should consider the overall PIP burden, rather than the simple presence or absence of PIPs. Moreover, it is critical to assess the severity of the overall histological activity over time, and the presence of other known risk factors for IBD in a given patient when we estimate the absolute risk in each IBD patient.

Data assessing the potential of malignant transformation of the PIPs themselves are scarce, and conventionally it is not suggested that PIPs be removed. Of note, they are usually removed or considered for surgery in the case of complications other than malignancy, such as bleeding or intussusception. Apart from case reports, only one recent study assessed and reported the presence of dysplasia in a relatively small-scale study [56,68,69]. Although the reported dysplasia frequency was relatively high, this requires confirmation. Practically speaking, it is important to recognize that the presence of innumerous PIPs can hinder the detection of dysplastic lesions or cancerous polyps during endoscopic surveillance. In addition, true cancerous polyps may resemble PIPs in the context of chronic colonic inflammation, potentially leading to missed diagnoses during colonoscopy. Physicians should exercise heightened vigilance when evaluating dysplastic lesions, as PIPs can obscure thorough assessment, increasing the risk of overlooking an adenomatous component within the PIP. Of note, the true risk is difficult to estimate, since the evidence is mainly limited to case reports.

Physicians should also be aware of other very rare solitary polyps, such as inflammatory cap polyps (non-cancerous polyp covered by a cap of fibropurulent exudates) [71], which may be present in few IBD patients and may be occasionally misclassified as PIPs.

Finally, an important practical comment is that the success of any surveillance technique depends on the quality of the bowel preparation. Achieving excellent bowel preparation may be challenging in some patients with IBD with pre-existing mucosal damage (e.g., stricturing disease or high PIP burden). Therefore, the treating physicians should pay attention to achieve optimal bowel preparation, to enable a high-quality surveillance colonoscopy irrespective of the endoscopic technique used [72].

In summary, controversy persists regarding the need for intensified surveillance strategies in IBD patients with a limited number of PIPs (in the absence of high PIP burden, and additional risk factors) and also whether targeted surveillance of the PIPs should be adopted as a routine practice.

## 6. Conclusions

In summary, there is an increasing body of evidence supporting an association between the presence of PIPs and risk of colorectal neoplasia. However, we should recognize that PIPs should be identified as a possible indicator of this risk through increased inflammation, and that their presence may be associated with a distinct patient phenotype, including the presence of other known risk factors in the same patients.

Further studies are needed to explore the inflammation and carcinogenesis pathways in patients with PIPs to better understand their potential role in carcinogenesis. These studies could provide valuable insights into the mechanisms by which PIPs contribute to CRC risk, helping to clarify their significance and guide more targeted surveillance strategies in the future.

Cancer surveillance in IBD patients with PIPs represents a unique challenge due to its possibly resource-intensive and time-consuming nature. Current guidelines generally recommend an intermediate surveillance interval of every three years when PIPs are present. However, evidence suggests that PIPs themselves may not warrant a more intensive surveillance strategy. Instead, the degree and extent of mucosal inflammation should guide surveillance frequency, with intervals of three years for mild to moderate inflammation and one year for moderate to severe inflammation. In the case of sustained mucosal healing, extending surveillance intervals beyond three years may be feasible. Advances in high-definition colonoscopy and the growing implementation of artificial intelligence (AI) hold promise for improving the detection of neoplastic lesions and differentiating adenomatous polyps from PIPs [73,74,75].

**Table 1:** Summary of the population of the reviewed publications.

Included Studies	Study Design	IBD Phenotypes	Region	PSC (n, %)	Extensive/Pan Colitis (n, %)	Median Follow-Up Time (Year)	Conclusion
Rutter et al. 2004 [30]	Case–control study	UC	Europe (UK)	NR	204 (100%)	NR	PIPs increased the risk of CRC.
Velayos et al. 2006 [22]	Case–control study	UC	America	50 (13.3%)	318 (84.6%)	NR	PIPs increased the risk of CRC.
Baars et al. 2011 [31]	Case–control study	UC, CD, Unclassified IBD	Europe	22 (4.3%)	156 (30.4%)	15.5 years	PIPs increased the risk of CRC.
Lutgens et al. 2015 [76]	Case–control study	UC, CD, Unclassified IBD	Europe	30 (5.7%)	349 (65.7%)	NR	PIPs increased the risk of CRC.
Jegadeesan et al. 2016 [77]	Case–control study	UC	America	47 (10.1%)	457 (97.9%)	3.0 years	PIPs did not increase the risk of CRN.
Choi et al. 2017 [78]	Cohort study	UC	Europe (UK)	42 (4.3%)	987 (100%)	13.0 years	PIPs did not increased the risk of CRC.
Jong et al. 2019 [65]	Cohort study	UC, CD, Unclassified IBD	Europe	27 (5.2%)	345 (66.5%)	21.6 years in PIPs, 22.9 years in non-PIPs.	PIPs did not increase the risk of CRN, ACRN or CRC.
Mahmoud et al. 2019 [32]	Cohort study	UC, CD, Unclassified IBD	Europe/America	238 (14.8%)	1275 (80.6%)	5.4 years in PIPs, 4.5 years in non-PIPs.	PIPs did not increase the risk of CRN or ACRN.
Xu et al. 2020 [79]	Cohort study	UC	Asia	10 (4.1%)	116 (47.2%)	13.0 years	PIPs increased the risk of ACRN.
Wolf et al. 2023 [62]	Case–control study	UC	Europe	10 (5.8%)	148 (85.5%)	4.8 years in PIPs, 5.5 years in non-PIPs	PIPs did not increase the Risk of CRN

**Table 2:** Clinical parameters reported to be associated with the presence of post-inflammatory polyps (PIPs).

Ulcerative colitis (rather than Crohn’s disease)
Extensive colitis
Higher inflammatory burden (severe colitis)
Longer disease duration
Primary sclerosing cholangitis
